# Implication of liver enzymes on incident cardiovascular diseases and mortality: A nationwide population-based cohort study

**DOI:** 10.1038/s41598-018-19700-8

**Published:** 2018-02-28

**Authors:** Kyung Mook Choi, Kyungdo Han, Sanghyun Park, Hye Soo Chung, Nam Hoon Kim, Hye Jin Yoo, Ji-A Seo, Sin Gon Kim, Nan Hee Kim, Sei Hyun Baik, Yong Gyu Park, Seon Mee Kim

**Affiliations:** 1 Division of Endocrinology and Metabolism, Department of Internal Medicine, College of Medicine, Korea University, Seoul, Korea; 2 Department of Biostatistics, College of Medicine, The Catholic University of Korea, Seoul, Korea; 3 Department of Family Medicine, College of Medicine, Korea University, Seoul, Korea

## Abstract

Although liver enzymes, such as γ-glutamyltransferase (GGT), alanine aminotransferase (ALT), and aspartate aminotransferase (AST), have recently been suggested as risk factors for cardiovascular diseases (CVD), impact on mortality after myocardial infarction (MI) or ischemic stroke (IS) was not previously examined. Using a population-based, nationwide cohort database, we explored the implication of GGT and aminotransferases on the development of CVD and all-cause mortality during a median 9.1 years of follow-up. Among 16,624,006 Korean adults, both GGT and aminotransferases exhibited a positive relationship with MI, IS, and mortality in a multivariate adjusted model. ALT and AST showed U-shaped associations with mortality, whereas GGT showed a positive linear relationship with mortality. The risk of 1-year mortality after MI or IS was significantly higher in the highest quartile of GGT compared to the lowest quartile (HR, 1.46; 95% CI, 1.40-1.52). The implication of GGT on MI, IS, and mortality persisted regardless of traditional cardiovascular risk parameters. This study demonstrated the unique pattern of association of ALT, AST, and GGT with the development of CVD and all-cause mortality in the Korean population. In particular, GGT showed the most robust linear relationship with mortality before and after cardiovascular events independent of risk factors.

## Introduction

Liver enzymes such as γ-glutamyltransferase (GGT), alanine aminotransferase (ALT), and aspartate aminotransferase (AST), have been used as markers of hepatic dysfunction and non-alcoholic fatty liver disease (NAFLD)^1^. These enzymes have attracted attention as emerging risk factors for cardiovascular disease (CVD), although there has been discordance in the relationship between specific liver enzymes and CVD^2^. Fraser *et al*. reported a meta-analysis exploring the association of GGT and ALT with incident coronary heart disease (CHD), stroke, and combined outcomes^3^. They found that GGT, but not ALT, was related to incident cardiovascular events independently of alcohol intake^3^. In another recent meta-analysis, the pooled adjusted relative risk (RR) for CVD was 1.23 (1.16–1.29) per standard deviation (SD) change in log baseline levels of GGT^4^. Conversely, a stratified analysis demonstrated that ALT is inversely associated with CHD and positively associated with stroke^4^. Zhang *et al*. reported the association between GGT and risk of stroke, although sex and ethnicity variations exist^5^. However, Unalp-Arida *et al*. recently reported that elevated ALT, AST, and GGT levels are unrelated to all-cause mortality and mortality from CVD, cancer, or diabetes, and GGT is only associated with increased all-cause mortality (hazard ratio [HR], 1.45; 95% confidence interval [CI], 1.21–1.74)^6^. In contrast, Lee *et al*. showed that elevated ALT and AST are associated with CVD mortality and all-cause mortality^7^. These variations in the findings of previous studies suggest the differential impact of specific liver enzymes on CVD and mortality. Furthermore, these discordant results may reflect small sample size, insufficient follow-up period, or differences in age, gender, and ethnicity. In a review investigating the relationship between liver enzymes, NAFLD, and incident CVD, Ghouri *et al*. concluded that biochemical and imaging markers of NAFLD are insufficient to identify patients at high risk for CVD^8^. Furthermore, to the best of author’s knowledge, there is no previous report about the impact of GGT and aminotransferases on mortality after the development of MI or stroke.

The National Health Insurance Sharing Service (NHISS) manages a population-based, nationwide cohort database with information about utilization of health insurance and periodic health examination for almost all Korean adults^9^.

In the present study, the implication of GGT and aminotransferases on CVD and all-cause mortality was evaluated using the longitudinal NHISS database, which includes more than 16,000,000 Korean men and women. In addition, the influence of GGT on 30-day, 90-day, and 1-year mortality after development of myocardial infarction (MI) and ischemic stroke (IS) was explored. Furthermore, the differential impact of GGT on the development of CVD and mortality according to risk factor subgroups was examined to explore interaction of confounding risk variables.

## Subjects and Methods

### Study design and participants

The Korean National Health Insurance Program is a government-operated mandatory social health insurance program that covers almost the entire (about 97%) Korean population. The remaining 3% of Korean people is covered by the Medical Aid program. The National Health Insurance System (NHIS) in Korea is composed of a comprehensive set of health information including around 50 million Koreans. The NHIS contains an eligibility database, health examination database, medical treatment database, and medical care institution database^9,10^. The NHISS, which includes claims and mortality data, is open to any researchers whose protocols are approved by the NHISS review committee. The Korea University institutional review board approved this study protocol in accordance with the Declaration of Helsinki of the World Medical Association.

NHIS recommends that all eligible Korean adults undergo a standardized health checkup every two years. Anthropometric and laboratory measurements including fasting glucose, lipid profile, creatinine, liver enzymes, and urinalysis are performed. Health-related behavioral variables, such as smoking, alcohol consumption, physical activity, and detailed medical history are assessed and recorded. Blood samples are taken after an overnight fast, and quality control procedures follow the Korean Association of Laboratory Quality Control. Data from a database of 16,094,237 Korean residents aged 20 years or older who had participated at least one biennial health examination provided by NHIS was examined.

### Definitions

MI, IS, and all-cause mortality were examined as primary outcomes. Diagnosis of MI was defined using the International Classification of Diseases, tenth revision (ICD-10) codes (I21-I22) and admission record. Diagnosis of IS was based on ICD-10 codes (I63-I64), CT or MRI claim data, and admission record. All-cause mortality was also examined. The health examination questionnaire categorized smoking status as “non-smokers, ex-smokers, or current smokers,” and alcohol drinking as “0, ≤2, 3–4, ≥5 times/week”. “Strenuous physical activity that was performed for at least 20 min” was defined as regular exercise, and subjects were graded as “exercising 0, 1–2, 3–4, ≥5 times/week”. Due to the change of questionnaire about physical activity after 2009, subjects were graded as “exercising 0, ≥1 times/week.” in analysis of mortality after MI or IS and forest plot analysis. Income level was dichotomized at the lower 10%. The presence of type 2 diabetes was defined based on criteria of fasting glucose level ≥7 mmol/L or the presence of at least one claim per year for a prescription of antidiabetic medication under ICD-10 codes (E11-E14). The presence of hypertension was defined based on criteria of systolic/diastolic blood pressure ≥140/90 mmHg or the presence of at least one claim per year for a prescription of antihypertensive agent under ICD-10 codes (I10–I13, I15). The presence of dyslipidemia was defined based on criteria of total cholesterol ≥6.21 mmol/L or the presence of at least one claim per year for a prescription of antihyperlipidemic agent under ICD-10 codes (E78).

### Statistical analysis

Data are expressed as mean ± standard deviation, geometric mean (95% confidence interval [CI]), or percentage. Differences between groups were identified using the Student’s t-test or χ^2^-test to assess differences in the distribution of categorical variables. Event incidence rates are presented as 1000 person-years. Cox proportional hazards regression analysis was used to evaluate the association between quartiles of ALT, AST, and GGT and the incidence of outcomes. Kaplan–Meier curves were plotted and compared with the log-rank test. A multiple Cox proportional hazard model was used after adjusting for age, sex, BMI, smoking status, alcohol consumption, exercise, diabetes, dyslipidemia, and hypertension. Forest plots for incident MI, IS, and mortality according to risk factor subgroups were evaluated. Interactions between variables were tested. All statistical results were analyzed using SAS 9.4 (SAS Institute Inc., Cary, NC, USA) and *P*-values < 0.05 were considered statistically significant. All statistical analyses were performed by an experienced professional statistician who is one of the authors (Han K.D., Ph.D.).

## Results

### Baseline characteristics of study subjects

Table 1 shows baseline characteristics of study subjects according to the quartiles of GGT levels. The higher quartile of the GGT group exhibited a higher proportion of male and elderly subjects compared to the lower quartile group. Furthermore, current smokers and heavy drinkers increased in the higher quartile of the GGT group. The prevalence of chronic metabolic diseases, including hypertension, type 2 diabetes, and dyslipidemia, also increased. As expected, ALT and AST concentrations showed stepwise incremental increases according to quartiles of GGT values.

**Table 1:** Baseline characteristics of study subjects according to the qurtiles of γ-glutamyltransferase (GGT).

	Q1	Q2	Q3	Q4	*P*
	(n = 4,078,542)	(n = 4,077,271)	(n = 3,924,511)	(n = 4,013,913)	
Age (years, %)	42.4 ± 15.0	44.4 ± 14.6	46.2 ± 14.0	48.4 ± 13.1	<0.001
20~39	1922952 (47.2)	1648984 (40.4)	1342441 (34.2)	1055911 (26.3)	
40~64	1757030 (43.1)	2002806 (49.1)	2152159 (54.8)	2487294 (62.0)	
≥65	398560 (9.8)	425481 (10.4)	429911 (11.0)	470708 (11.7)	
Sex, n (%)					<0.001
Male	2074945 (50.9)	2151451 (52.8)	2219904 (56.6)	2146591 (53.5)	
Female	2003597 (49.1)	1925820 (47.2)	1704607 (43.4)	1867322 (46.5)	
Height (cm)	164 ± 9.0	163.8 ± 9.1	163.8 ± 9.2	162.8 ± 9.3	<0.001
Weight (kg)	60.2 ± 9.9	62.1 ± 10.8	64.6 ± 11.7	66.1 ± 12.2	<0.001
BMI (kg/m^2^)	22.3 ± 2.7	23.1 ± 3.0	24.0 ± 3.1	24.8 ± 3.3	<0.001
SBP (mmHg)	118.7 ± 14.8	121.1 ± 15.3	123.9 ± 15.8	127.4 ± 16.6	<0.001
DBP (mmHg)	73.9 ± 9.8	75.6 ± 10.1	77.5 ± 10.4	79.7 ± 10.8	<0.001
FPG (mg/dL)	90.7 ± 17.1	92.6 ± 19.7	95.6 ± 23.3	101.2 ± 29.3	<0.001
Total cholesterol (mg/dL)	179.8 ± 32.3	189.0 ± 33.9	196.8 ± 35.6	204.8 ± 39.1	<0.001
ALT (IU/L)	15 (12, 20)	18 (14, 24)	22 (16, 30)	29 (20, 43)	<0.001
AST (IU/L)	20 (17, 24)	21 (18, 25)	23 (19, 28)	27 (22, 35)	<0.001
GGT (IU/L)	12 (10, 16)	21 (14, 25)	32 (19, 40)	58 (32, 86)	<0.001
Smoking status, n (%)					<0.001
Never smoker	2942431 (72.1)	2793523 (68.5)	2496120 (63.6)	2461028 (61.3)	
Ex-smoker	307776 (7.6)	333532 (8.2)	356031 (9.1)	336887 (8.4)	
Current smoker	828335 (20.3)	950216 (23.3)	1072360 (27.3)	1215998 (30.3)	
Alcohol drinking, n (%)					<0.001
None	2426279 (59.5)	2215856 (54.4)	1909132 (48.7)	1780663 (44.4)	
≤2 times a week	1514272 (37.1)	1622970 (39.8)	1628249 (41.5)	1524607 (38.0)	
3-4 times a week	100732 (2.5)	173956 (4.3)	276387 (7.0)	464979 (11.6)	
≥5 times a week	37259 (0.9)	64489 (1.6)	110743 (2.8)	243664 (6.1)	
Exercise, n (%)					<0.001
None	2239639 (54.9)	2224361 (54.6)	2109130 (53.7)	2197428 (54.8)	
1-2 times a week	1082238 (26.5)	1091760 (26.8)	1082421 (27.6)	1097387 (27.3)	
3-4 times a week	438499 (10.8)	440089 (10.8)	422498 (10.8)	404055 (10.1)	
≥5 times a week	318166 (7.8)	321061 (7.9)	310462 (7.9)	315043 (7.9)	
Income (lower 20%, %)	385285 (9.5)	384897 (9.4)	371010 (9.5)	409198 (10.2)	<0.001
Diabetes, n (%)	146306 (3.6)	205985 (5.1)	296818 (7.6)	512554 (12.8)	<0.001
Hypertension, n (%)	603160 (14.8)	827703 (20.3)	1062950 (27.1)	1478801 (36.8)	<0.001
Dyslipidemia, n (%)	243235 (6.0)	408302 (10.0)	596101 (15.2)	931049 (23.2)	<0.001

BMI, body mass index; SBP, systolic blood pressure; DBP, diastolic blood pressure; FPG, fasting plasma glucose; TC, total cholesterol; ALT, alanine aminotransferase; AST, aspartate aminotransferase.

### Implications of ALT, AST, and GGT in MI, IS, and all-cause mortality

**Figure 1:**
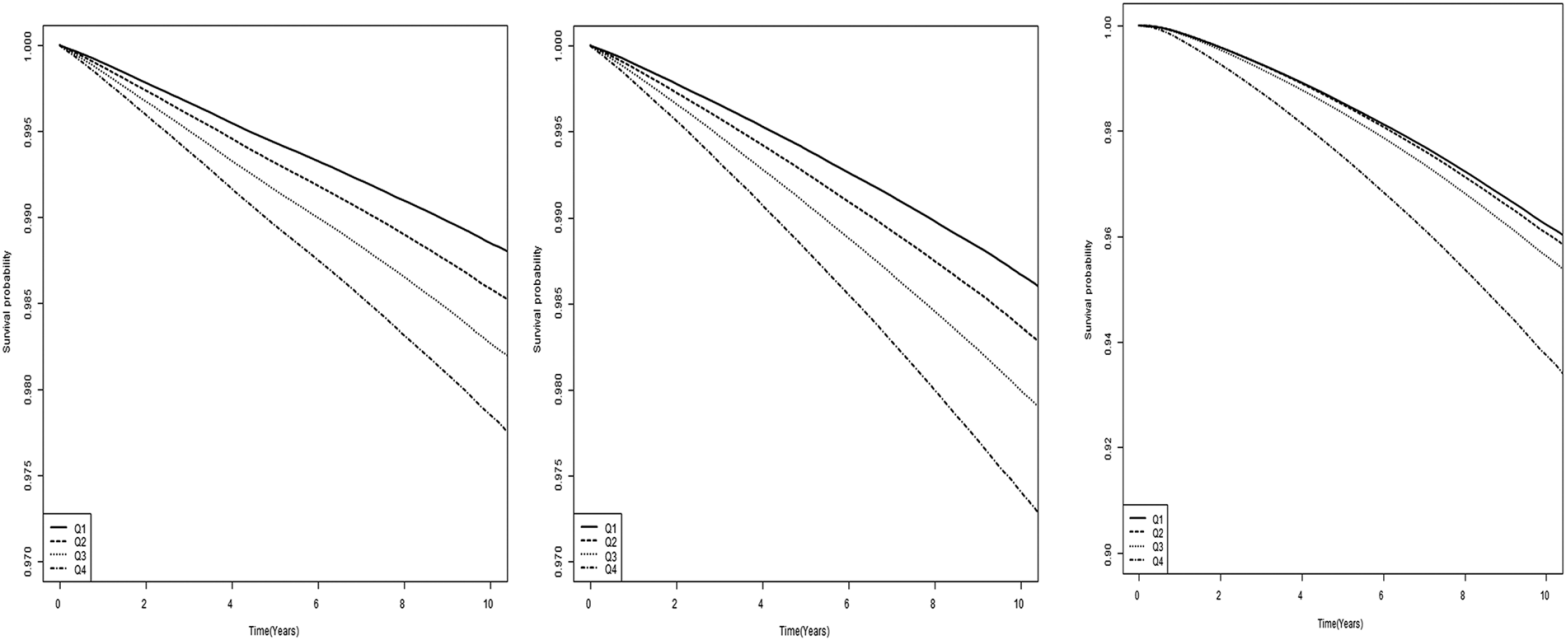
Kaplan-Meier survival curves for freedom from myocardial infarction, ischemic stroke, and all-cause mortality according to quartiles of γ-glutamyltransferase (GGT). (**A**) Myocardial infarction. (**B**) Ischemic stroke. (**C**) Mortality.

In Table 2, multivariate analysis adjusted for age, sex, BMI, smoking, alcohol, exercise, diabetes, hypertension, and dyslipidemia demonstrates the influence of GGT and aminotransferases on development of MI, IS, and death in Korean men and women. The adjusted HR of MI was 1.10 (95% CI, 1.07–1.10; *P* < 0.001) in the highest quartile of ALT and 1.05 (95% CI, 1.04–1.06; *P* < 0.001) in the highest quartile of AST compared to their lowest quartile counterparts, respectively. A much stronger relationship between GGT and MI was found, with adjusted HR of 1.27 (95% CI, 1.26–1.29; *P* < 0.001). For IS and all-cause mortality, a similar association with ALT, AST, and GGT was observed, and GGT was the strongest risk indicator (IS: HR, 1.36; 95% CI, 1.34–1.37; *P* < 0.001; Mortality: HR, 1.64; 95% CI, 1.63–1.66; *P* < 0.001). Interestingly, both ALT and AST revealed U-shaped associations with mortality, whereas GGT demonstrated a positive linear relationship. Kaplan-Meier survival curves for MI, IS, and mortality showed clear differences and dispersion with time passage according to the quartiles of GGT (Fig. 1). The highest GGT group exhibited the worst survival as well as a higher incidence of MI and IS.

**Table 2:** Hazard ratios and 95% confidence intervals for myocardial infarction, ischemic stroke, and death according to quartiles of liver enzymes.

		Myocardial infarction				Ischemic stroke				Death			
		Event	Duration	IR	HR (95% Cl)	Event	Duration	IR	HR (95% Cl)	Event	Duration	IR	HR (95% Cl)
ALT	Q1	48700	36870026	1.32	1.00 (ref.)	59161	36847085	1.61	1.00 (ref.)	179742	37046039	4.85	1.00 (ref.)
	Q2	54532	35893859	1.52	1.00 (0.99, 1.01)	65599	35869654	1.83	1.00 (0.99, 1.01)	154118	36102372	4.27	0.89 (0.89, 0.90)
	Q3	58359	34273199	1.70	1.02 (1.01, 1.03)	68356	34255343	2.00	1.02 (1.01, 1.03)	147344	34501798	4.27	0.91 (0.91, 0.92)
	Q4	69993	37444966	1.87	1.10 (1.07, 1.10)	77226	37438130	2.06	1.06 (1.05, 1.07)	177221	37720098	4.70	1.17 (1.17, 1.18)
AST	Q1	40948	34854743	1.17	1.00 (ref.)	45806	34849351	1.31	1.00 (ref.)	110512	35011148	3.16	1.00 (ref.)
	Q2	55205	38434636	1.44	0.97 (0.96, 0.98)	61766	38427855	1.61	0.95 (0.94, 0.96)	143560	38648886	3.71	0.91 (0.90, 0.92)
	Q3	62827	37579639	1.67	0.97 (0.96, 0.98)	74750	37556028	1.99	0.96 (0.95, 0.97)	166036	37823728	4.39	0.95 (0.94, 0.95)
	Q4	72604	33613032	2.16	1.05 (1.04, 1.06)	88020	33576979	2.62	1.04 (1.03, 1.05)	238317	33886545	7.03	1.30 (1.30, 1.31)
GGT	Q1	42421	36982585	1.15	1.00 (ref.)	48844	36974336	1.32	1.00 (ref.)	138441	37142806	3.73	1.00 (ref.)
	Q2	51835	36735422	1.41	1.08 (1.06, 1.09)	59570	36723544	1.62	1.09 (1.08, 1.11)	143240	36934518	3.88	1.05 (1.04, 1.06)
	Q3	60810	35233338	1.73	1.16 (1.15, 1.17)	70169	35217336	1.99	1.20 (1.18, 1.21)	153119	35470795	4.32	1.15 (1.14, 1.16)
	Q4	76518	35530706	2.15	1.27 (1.26, 1.29)	91759	35494998	2.59	1.36 (1.34, 1.37)	223625	35822188	6.24	1.64 (1.63, 1.66)

IR, incidence rate per 1000.

Hazard ratio was analyzed after adjusting for age, sex, body mass index, smoking, alcohol, exercise, diabetes, hypertension, and dyslipidemia.

### Implications of ALT, AST, and GGT in mortality after the development of MI or IS

**Figure 2:**
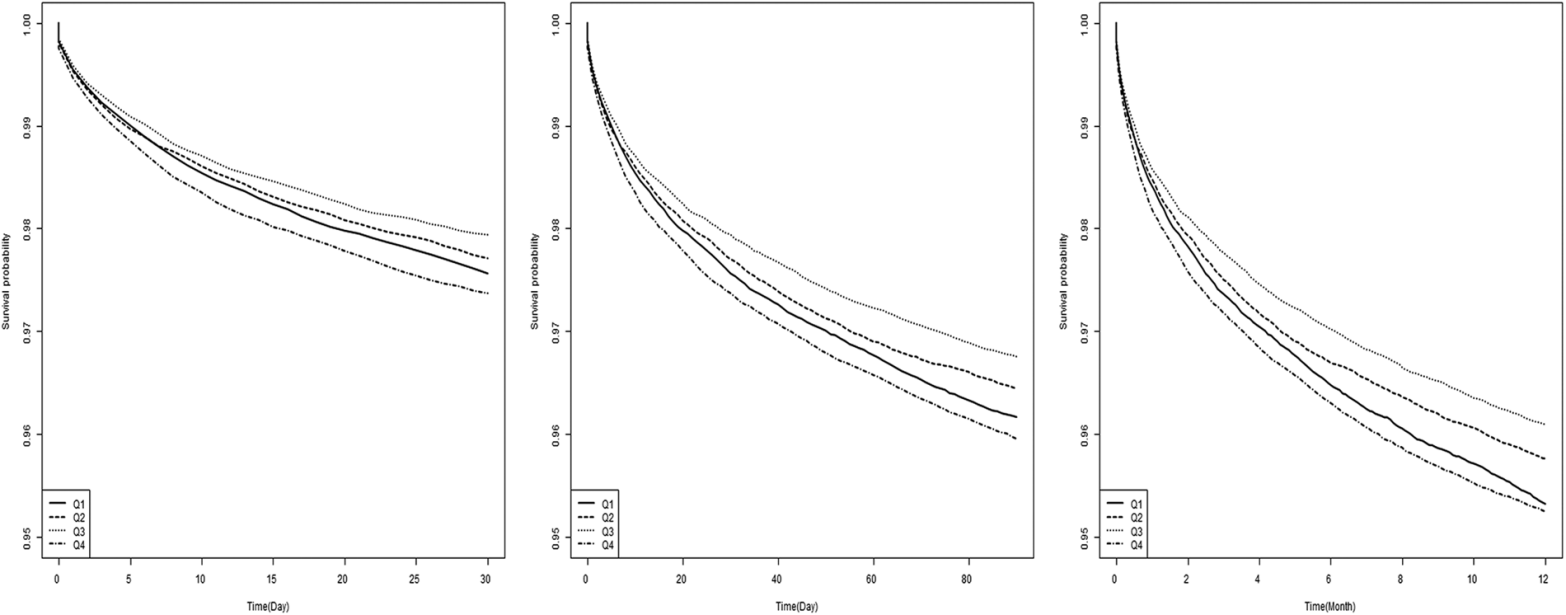
Kaplan-Meier survival curves for freedom from 30-day, 90-day, and 1-year mortality according to γ-glutamyltransferase (GGT) quartiles after myocardial infarction or ischemic stroke. (**A**) 30-day mortality. (**B**) 90-day mortality. (**C**) 1-year mortality.

Table 3 demonstrates the association of liver enzymes with 1-year mortality after the development of MI or IS after adjusting for confounding factors. Consistent with our previous results, ALT and AST showed similar U-shaped associations with mortality, whereas GGT revealed a positive linear relationship. The risk of all-cause mortality after MI or IS was 46% higher in the highest quartile of GGT compared to the lowest quartile counterparts (HR, 1.46; 95% CI, 1.40–1.52). In Kaplan-Meier survival analysis, quartile groups of GGT exhibited different 30-day, 90-day, and 1-year survival after the development of CVD, showing increased mortality in the higher GGT groups (Fig. 2).

**Table 3:** Hazard ratios and 95% confidence intervals for mortality according to quartiles of liver enzymes after myocardial infarction or ischemic stroke.

		Event	Duration	MR	Model 1	Model 2
					HR (95% Cl)	HR (95% Cl)
ALT	Q1	6218	21507006	0.29	1.00 (Ref)	1.00 (Ref)
	Q2	3841	20805510	0.18	0.73 (0.70, 0.76)	0.80 (0.76, 0.83)
	Q3	3571	22471487	0.16	0.70 (0.67, 0.73)	0.80 (0.77, 0.84)
	Q4	3252	21550048	0.15	0.78 (0.74, 0.81)	0.93 (0.89, 0.97)
AST	Q1	5024	23428822	0.21	1.00 (Ref)	1.00 (Ref)
	Q2	3378	18599784	0.18	0.83 (0.80, 0.87)	0.87 (0.83, 0.91)
	Q3	3929	22604257	0.17	0.80 (0.77, 0.83)	0.85 (0.82, 0.89)
	Q4	4551	21702283	0.21	1.02 (0.98, 1.06)	1.09 (1.04, 1.13)
GGT	Q1	4622	20642384	0.22	1.00 (Ref)	1.00 (Ref)
	Q2	4061	21699165	0.19	0.93 (0.89, 0.97)	1.03 (0.99, 1.07)
	Q3	3740	22372024	0.17	0.92 (0.88, 0.96)	1.08 (1.04, 1.13)
	Q4	4459	21619018	0.21	1.24 (1.19, 1.29)	1.46 (1.40, 1.52)

MR, mortality rate (per 1000 person-years); HR, hazard ratio; CI, confidence interval; ALT, alanine aminotransferase; AST, aspartate aminotransferase; GGT, γ-glutamyltransferase.

Model 1: HRs were analyzed after adjusting for age and sex.

Model 2: HRs were analyzed after adjusting for age, sex, body mass index, smoking, alcohol, exercise, diabetes, hypertension, and dyslipidemia.

### Interactions of anthropometric, lifestyle, and chronic disease factors

**Figure 3:**
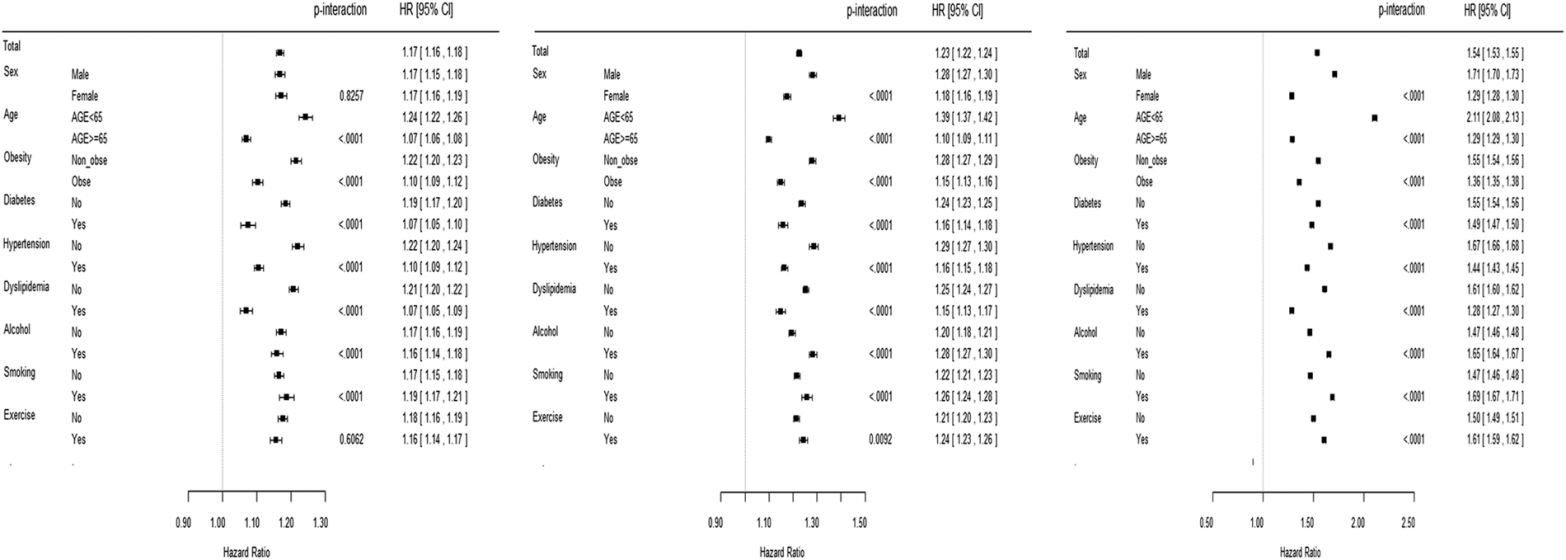
Forest plot subgroup analysis of myocardial infarction, ischemic stroke, and all-cause mortality across highest quartile values of γ-glutamyltransferase (GGT). (**A**) Myocardial infarction. (**B**) Ischemic stroke. (**C**) Mortality.

A forest plot in Fig. 3 shows interaction by age, sex, BMI, diabetes, hypertension, dyslipidemia, smoking, alcohol consumption, and exercise. Several previous studies have shown interactions of age, sex, and other factors, and the present study demonstrated these interactions across the highest quartile values of GGT levels. Individuals <65 years of age demonstrated remarkably increased risk of MI, IS, and mortality associated with GGT compared to those ≥ 65 years. Furthermore, other confounding factors including sex, metabolic diseases, and lifestyle factors affect the relationship of GGT with CVD and all-cause mortality. However, the increased risk of MI according to GGT levels was similar between men and women (*P*-interaction = 0.826). Importantly, the significant relationship of GGT with MI, IS, and mortality persisted independent of other risk factors.

## Discussion

In this cohort study including a huge Korean population participating in a regular public health examination program, GGT showed a positive linear relationship with risk of CVD and all-cause mortality, whereas both ALT and AST had U-shaped associations with mortality during a median 9.1 years of follow-up. Overall, GGT had a more resolute influence on CVD and mortality compared to aminotransferases. Moreover, this study first demonstrated that higher GGT levels reflect increased risk of 1-year mortality after the development of MI or IS after adjusting for conventional risk factors.

Previous studies have suggested a potential link between NAFLD and CVD. Lee *et al*. reported the relationship between severity of NAFLD and estimated 10-year CVD risk using the pooled cohort equation^11^. Accumulation of liver fat and elevated liver enzymes are associated with type 2 diabetes and hypertension^12,13^, both of which are representative risk factors for CVD. We observed the synergistic impact of NAFLD and metabolic syndrome on subclinical atherosclerosis^14^. Furthermore, using ^18^F-fluorodeoxyglucose positron emission tomography (FDG-PET), we found that NAFLD is associated with vascular inflammation, which may reflect rupture-prone vulnerable atherosclerotic plaques^15^. Both visceral fat accumulation and risk of metabolic syndrome are significantly correlated with GGT and ALT levels^16,17^. Liver enzyme assays are a low-cost, simple, sensitive, and standardized method, which may support to assess cardiovascular risk^4^.

ALT is mainly produced by the liver due to increased hepatic inflammation or injury, whereas AST originates from liver and muscle cells and rises with myocardial cell injury or hepatic dysfunction^2^. There is a significant amount of data linking liver enzymes and incident diabetes. In a recent meta-analysis, a HR of 1.85 is related to 1 logged IU/L increment of ALT, whereas HR of 1.92 is associated with 1 logged IU/L increment of GGT after adjustment for important diabetes risk factors^12^. Moreover, mild NAFLD diagnosed using ultrasonography was found to be a determinant of incident diabetes^12^. However, the association between liver transaminase enzymes and CVD has been debated. The association between baseline ALT levels and CVD events was attenuated in multivariate models in the Framingham Offspring Study^18^. In a Korean study of 37,085 patients who underwent health examination at a hospital, elevated ALT levels were associated with increased CVD- or diabetes-related mortality^19^. However, there was no subgroup analysis using the non-diabetic group for CVD-related mortality. In a study of 455 community dwelling elderly men, Elinav *et al*. showed that low ALT levels are associated with increased long-term mortality^20^. Recently, Oh *et al*. reported a U-shaped relationship between serum ALT concentrations and all-cause death in individuals aged ≥ 60 years^21^. However, a meta-analysis failed to demonstrate a reliable relationship between ALT and CHD or stroke^3^. Decisively, a review based on prospective data summarized that current evidence does not support the linear relationship between ALT and CVD events^8^. The present study including an enormous Korean population provides evidence for the relationship of ALT and AST with CVD and mortality, although GGT was more closely associated with CVD or mortality than aminotransferases. Interestingly, the present study demonstrated a U-shaped relationship between aminotransferases and all-cause mortality. The difference in the individual mechanism of GGT and aminotransferases has not been clearly elucidated, and further research is needed.

Several prospective studies have reported an association between GGT and development of cardiovascular events independent of alcohol intake. In a study of 7,613 middle-aged British men, Wannamethee *et al*. reported that GGT levels were positively associated with all-cause mortality, mortality from ischemic heart disease, and cardiovascular risk factors such as BMI, total cholesterol, and diabetes^22^. In another longitudinal study, GGT was an independent risk factor for cardiovascular mortality in 163,944 Austrian adults^23^. Interestingly, higher GGT was associated with chronic forms of coronary heart disease and stroke, but not AMI in men^23^. In 2007, Lee *et al*. showed the longitudinal relationship between GGT and metabolic syndrome, CVD, and mortality in the Framingham Offspring Study^24^. They found that a 1-SD elevation of log-GGT is associated with a 13% higher risk of CVD after adjusting for established CVD risk factors^24^. In a prospective study of 6,997 men, risk of coronary heart disease and CVD mortality was elevated in the highest quartile of GGT, whereas risk of stroke showed only a tendency of increased risk^25^. In a meta-analysis of 10 prospective studies, 1 IU/L higher GGT was associated with a HR = 1.20 (95% CI, 1.02–1.40) for CHD and HR = 1.54 (95% CI, 1.20–2.00) for stroke^3^. However, previous studies showed considerable heterogeneity. The present study includes the largest number of subjects and demonstrated that GGT showed a positive linear relationship with CVD and all-cause mortality even after adjusting for confounding factors. Furthermore, the Kaplan-Meir survival graph exhibits clear separation and dispersion of the survival curves of MI, IS, and mortality after the passage of time according to GGT quartiles. Interestingly, the present study demonstrated an increased risk of 1-year mortality according to higher GGT levels after MI or IS.

GGT has been established as a sensitive biomarker of liver function that reflects NAFLD, hepatitis, and alcohol ingestion^2^. Previous studies demonstrated a close relationship between GGT and hepatic triglyceride accumulation in obesity, insulin resistance, metabolic syndrome, and type 2 diabetes^26,27^. Recent studies have suggested that hepatokines, predominantly liver-derived proteins, may link obesity, metabolic syndrome, and CVD^28^. As mediating mechanisms, GGT has been known as a proinflammatory indicator and a marker of oxidative stress^2^. Kawamoto *et al*. reported the synergistic effects of higher high-sensitivity C-reactive protein (hsCRP) and GGT levels on metabolic syndrome and insulin resistance in the general population^29^. GGT is the enzyme responsible for catabolism of glutathione, the major antioxidant in humans^30^. Moreover, GGT impacts LDL cholesterol oxidation to influence plaque progression and rupture^31^. Paolicchi *et al*. found active GGT staining in human coronary atherosclerotic plaques^32^. They suggested the pathogenic role of GGT as an independent and synergistic factor in addition to conventional cardiovascular risk parameters. Although exact mechanism is not clear, the association between GGT and lipoproteins suggests that LDL lipoprotein can convey GTT activity inside the atherosclerotic plaque^31,33^. The GTT-mediated oxidative stress may have a role in the development and vulnerability of plaque, such as plaque erosion and rupture, enhance plate aggregation, and thrombosis^31,34^.

Previous studies have shown the close relationship between GGT and surrogate markers of CVD. Celik *et al*. reported significantly higher GGT levels in patients with coronary plaque than in controls using coronary computed tomography angiography (CCTA)^35^. In the Multi-ethnic Study of Atherosclerosis (MESA) study, continuous strong, positive associations between GGT and CRP, interleukin-6 (IL-6), and soluble intercellular adhesion molecule-1 (sICAM-1) were observed in multivariable models^36^. Cho *et al*. demonstrated the independent association between elevated serum GGT levels and coronary artery calcification (CAC) progression^37^. Recently, low bilirubin and high GGT were reported as potential biomarkers for coronary atherosclerosis in Korean men^38^.

Several studies have suggested that age influences the effect of GGT on CVD and mortality. In a nested case-control study, the association between GGT and incident CVD mortality was not found in subjects aged more than 70 years^39^. Similarly, Strasak *et al*. reported that a stronger association between GGT change and CVD mortality was observed in younger participants^40^. Wannamethee *et al*. also showed a stronger association between GGT and CVD mortality in younger men (<55 years) when stratified by age group^25^. However, Mahady *et al*. demonstrated that neither ALT nor GGT was associated with increased risk of all-cause and cardiovascular mortality in a younger age group (≤59 years), but both were associated with excess risk in older individuals^41^. The present study confirmed that age is involved in the relationship between liver enzymes, CVD, and mortality. Furthermore, our study revealed that individuals with age <65 demonstrated a remarkably higher risk of MI, IS, and mortality associated with GGT compared to those with age ≥65. On the other hand, a previous Austrian and British study did not find an association between GGT and stroke in women^3,23^, whereas a Japanese study reported a positive relationship only in women^42^. In this study, men exhibited a higher risk of IS and mortality associated with GGT than women, whereas the risk of MI was similar between men and women. Other cardiovascular risk factors, such as BMI, diabetes, hypertension, and dyslipidemia, also showed significant interactions in the association between GGT, CVD and mortality. However, their relationship was maintained regardless of the existence of risk factors. Consistent with our results, in a study including 17,852 adults from three British cohorts, higher GGT levels are associated with CVD mortality in both individuals with and without diabetes^43^. GGT has been used as a sensitive biomarker of alcohol consumption as well as hepatic inflammation and NAFLD. Subgroup analysis exhibited a persistent association of GGT with CVD and mortality independent of alcohol consumption, consistent with the results of previous studies^3,44^.

Dyslipidemia represents a major risk factor in CVD and small, dense LDL in nonalcoholic steatohepatitis (NASH) may increase risk for atherosclerosis and CVD^45^. Statins are most effective and commonly described drugs for dyslipidemia, although statin intolerance limits effective treatment^46^. Furthermore, statin treatment is usually tolerable in patients with chronic liver disease such as NAFLD and stable viral hepatitis^47^. Recently, approaches to minimize cardiovascular risk among patients with statin intolerance are recommended^48^. The potential link between statins, liver enzymes, and CVD might be needed to consider. MI, IS, and mortality according to statin usage at baseline are shown in Supplementary Table 1. Although statin therapy decreases the risk of outcome variables, interaction between statin therapy and liver enzymes was not found.

This study has some limitations. First, although we tried to adjust for multiple covariates to influence CVD and mortality, it was impossible to eliminate residual or unmeasured confounding factors. Second, only serum liver enzyme concentrations and other variables measured at baseline were analyzed. Third, despite exclusion of patients with medical histories of liver disease or diagnosis and treatment based on Korean insurance claim data, a few patients with rare causes of liver disease may have been included. However, our study has its own unique strengths. The present study included the largest amount of subjects ever, with more than 16,000,000 subjects and a follow-up period of 9.1 years. In addition, this study was performed based on a credible database covering almost the entire Korean population and including socio-demographic variables, laboratory information, medical diagnosis, medication, and mortality data. Therefore, comprehensive analyses regarding the effects of GGT, ALT, and AST on incident MI, IS, and mortality as well as subgroup analyses are possible after adjustment for important confounding factors.

## Conclusions

The present study demonstrated that liver enzymes, in particular GGT, are associated with long-term risk of CVD and mortality independent of other established CVD risk factors. GGT might be a simple and practical tool for the assessment of individual risk of CVD and mortality, and may provide a basis for the appropriate individual intervention to prevent CVD and mortality.

### Availability of data and materials

The data that support the findings of this study are available from the corresponding author upon reasonable request and approval of the Institutional Review Board.

## Electronic supplementary material

Supplementary Table
