# Supplementary material for: Implication of liver enzymes on incident cardiovascular diseases and mortality: A nationwide population-based cohort study
Source: Sci Rep. 2018 Feb 28;8:3764. doi: 10.1038/s41598-018-19700-8 (PMC5830612; doi:10.1038/s41598-018-19700-8)
Supplement: Supplementary file 1 — Supplementary Table [file 41598_2018_19700_MOESM1_ESM.pdf]

Original Article

Implication of liver enzymes on incident cardiovascular diseases and mortality: A nationwide population-based cohort study

Kyung Mook Choi[1], Kyungdo Han[2], Sanghyun Park[2], Hye Soo Chung[1], Nam Hoon Kim[1], Hye Jin Yoo[1], Ji-A Seo[1], Sin Gon Kim[1], Nan Hee Kim[1], Sei Hyun Baik[1], Yong Gyu Park[2*] and Seon Mee Kim[3*]

[1]Division of Endocrinology and Metabolism, Department of Internal Medicine, College of Medicine, Korea University, Seoul, Korea; [2]Department of Biostatistics, College of Medicine, The Catholic University of Korea, Seoul, Korea; [3]Department of Family Medicine, College of Medicine, Korea University.

Supplementary Table 1. Hazard ratios and 95% confidence intervals for myocardial infarction, ischemic stroke, and death according to quartiles of liver enzymes in non-statin and statin group

Non-statin group													
		Myocardial infarction				Ischemic stroke				Death			
		Event	Duration	IR	HR(95% CI)	Event	Duration	IR	HR(95% CI)	Event	Duration	IR	HR(95% CI)
ALT	Q1	33447	33689323.75	0.99	1(ref.)	42965	33679036.02	1.28	1(ref.)	133790	33759244.17	3.96	1(ref.)
	Q2	34425	31039672.97	1.11	0.998(0.983-1.013)	44781	31029352.45	1.44	1.015(1.001-1.028)	107585	31113362.56	3.46	0.895(0.888-0.902)
	Q3	34724	28203468.24	1.23	1.037(1.021-1.052)	44166	28194915.15	1.57	1.046(1.032-1.060)	98118	28275754.22	3.47	0.927(0.919-0.934)
	Q4	38036	28697499.85	1.33	1.138(1.120-1.156)	45954	28692485.11	1.6	1.103(1.087-1.118)	117280	28776178.6	4.08	1.298(1.287-1.309)
AST	Q1	25800	31303149.81	0.82	1(ref.)	30567	31300569.11	0.98	1(ref.)	76393	31356501.48	2.44	1(ref.)
	Q2	34534	33303831.25	1.04	0.984(0.969-1.001)	41732	33299012.22	1.25	0.968(0.954-0.983)	99786	33375492.95	2.99	0.920(0.912-0.929)
	Q3	37864	30952226.08	1.22	1.000(0.984-1.016)	49102	30941296.28	1.59	0.988(0.973-1.002)	113237	31032664.65	3.65	0.970(0.961-0.979)
	Q4	42434	26070757.66	1.63	1.112(1.094-1.130)	56465	26054911.13	2.17	1.085(1.070-1.101)	167357	26159880.46	6.40	1.421(1.409-1.433)
GTP	Q1	29849	33848369.86	0.88	1(ref.)	36284	33845293.42	1.07	1(ref.)	105630	33918254.45	3.11	1(ref.)
	Q2	33274	32110943.24	1.04	1.079(1.062-1.096)	41211	32102168.5	1.28	1.102(1.086-1.118)	101162	32180860.78	3.14	1.051(1.042-1.060)
	Q3	35975	29063718.76	1.24	1.173(1.155-1.192)	45315	29053603.02	1.56	1.214(1.197-1.231)	102254	29135191.95	3.51	1.173(1.163-1.184)
	Q4	41534	26606932.94	1.56	1.331(1.310-1.353)	55056	26594723.81	2.07	1.409(1.389-1.429)	147727	26690232.36	5.53	1.791(1.775-1.806)
Statin group													
		Myocardial infarction				Ischemic stroke				Death			
		Event	Duration	IR	HR(95% CI)	Event	Duration	IR	HR(95% CI)	Event	Duration	IR	HR(95% CI)
ALT	Q1	3588	598728.23	5.99	1(ref.)	3594	599964.17	5.99	1(ref.)	8723	612122.09	14.25	1(ref.)
	Q2	4991	1001047.82	4.99	0.918(0.879-0.959)	4868	1003478.81	4.85	0.907(0.868-0.947)	9666	1020969.59	9.47	0.825(0.801-0.849)
	Q3	6240	1416585.5	4.40	0.855(0.820-0.891)	6074	1419837.89	4.28	0.866(0.830-0.903)	11091	1442050.04	7.69	0.774(0.752-0.796)
	Q4	9627	2326296.39	4.14	0.878(0.843-0.914)	9219	2333019.7	3.95	0.890(0.855-0.927)	15372	2366458.39	6.50	0.824(0.802-0.848)
AST	Q1	3940	720531.71	5.47	1(ref.)	3810	722185.02	5.28	1(ref.)	7423	735745.81	10.09	1(ref.)

	Q2	5249	1096878.69	4.79	0.901(0.865-0.939)	4783	1100852.22	4.34	0.859(0.823-0.896)	9217	1118068.07	8.24	0.853(0.827-0.880)
	Q3	6666	1550128.91	4.30	0.856(0.823-0.891)	6512	1553734.68	4.19	0.851(0.817-0.886)	11635	1577542.75	7.38	0.843(0.818-0.868)
	Q4	8591	1975118.62	4.35	0.873(0.840-0.907)	8650	1979528.65	4.37	0.890(0.856-0.925)	16577	2010243.47	8.25	0.984(0.957-1.012)
GTP	Q1	2994	615302.07	4.87	1(ref.)	2728	617998.83	4.41	1(ref.)	6043	627321.27	9.63	1(ref.)
	Q2	4670	993569.53	4.70	1.007(0.962-1.054)	4333	996879.42	4.35	1.045(0.996-1.096)	8648	1012375.05	8.54	1.013(0.980-1.047)
	Q3	6706	1436217.16	4.67	1.048(1.003-1.095)	6301	1441033.56	4.37	1.113(1.063-1.165)	11470	1463888.8	7.84	1.058(1.025-1.092)
	Q4	10076	2297569.18	4.39	1.074(1.029-1.121)	10393	2300388.76	4.52	1.240(1.187-1.296)	18691	2338014.98	7.99	1.312(1.272-1.353)

IR, incidence rate per 1000

Hazard ratio was analyzed after adjusting for age, sex, body mass index, smoking, alcohol, exercise, diabetes, and hypertension.

Patients who are taking statin therapy during follow-up period are regarded as drop-out cases.
